# The Effect of Sport Motivation on Burnout in Adolescent Athletes—Chain Mediating Effect of Life Satisfaction and Mental Toughness

**DOI:** 10.5114/jhk/201433

**Published:** 2025-10-01

**Authors:** Hongli Zhang, Hongtao Ma

**Affiliations:** 1 College of Education, Beijing Sport University, Beijing, China.

**Keywords:** adolescent athletes, sport motivation, athlete burnout, life satisfaction, mental toughness

## Abstract

This study explored the effects of sport motivation on athlete burnout among adolescent athletes and examined the mediating roles of life satisfaction and mental toughness. A total of 638 adolescent athletes (mean age = 15.64 ± 2.28 years) were assessed using the athletic motivation scale, the satisfaction with life scale, the adolescent mental toughness scale, and the athlete burnout questionnaire. Data were analyzed using SPSS Process for chain mediation analysis. Results indicated that sport motivation negatively predicted athlete burnout in both male (r = −0.523) and female athletes (r = −0.491). For male athletes, the total effect of sport motivation on athlete burnout was −0.374, with direct and indirect effects of −0.188 and −0.186, respectively. For female athletes, the total effect was −0.601, with direct and indirect effects of −0.363 and −0.238, respectively. Male athletes showed indirect effects through mental toughness (effect = −0.174; share = 46.52%) and the chain mediation of life satisfaction and mental toughness (effect = −0.072; share = 19.25%). Female athletes exhibited indirect effects through life satisfaction (effect = −0.043; share = 7.15%), mental toughness (effect = −0.151; share = 25.12%), as well as the chain mediation of life satisfaction and mental toughness (effect = −0.044; share = 7.32%). This study highlights the role of sport motivation in reducing athlete burnout. For female athletes, burnout was influenced by all three mediating pathways (life satisfaction, mental toughness, and their chain mediation). For male athletes, the effects were predominantly mediated through mental toughness and the chain mediation of life satisfaction along with mental toughness.

## Introduction

Athlete burnout has become an increasingly prominent and challenging issue in competitive sports in recent years (Gray et al., 2023; Gustafsson et al., 2017). Under the high-intensity and high-pressure environment, athletes not only have to cope with the intrinsic pressure brought by training and competition, but also need to deal with the extrinsic pressure such as social expectations and media attention (Gustafsson et al., 2017). The prolonged accumulation of these pressures, if not effectively managed, can deplete mental and physical resources, ultimately leading to athlete burnout (Gustafsson et al., 2017). Recent research by Park et al. (2023) further highlights the role of effort-reward imbalance in exacerbating burnout among athletes, emphasizing the importance of balancing effort and perceived rewards to mitigate burnout (Park et al., 2023). This phenomenon is usually characterized by emotional or physical fatigue, a reduced sense of achievement and negative attitudes towards sport (Martinent et al., 2020). Athlete burnout is centred on energy depletion (Gustafsson et al., 2016; Maslach et al., 2001), the consequences of which include decreased sport motivation, diminished mental toughness, and may even lead to an early end of a sporting career (Gustafsson et al., 2014). Previous studies have indicated that physical activity and social support play a significant role in mitigating athlete burnout (Gustafsson et al., 2011), but in recent years, researchers have begun to focus on the interventional role of mental factors such as mental toughness and life satisfaction (Curran et al., 2013). Mental toughness plays an important role in this process as an internal resource that helps athletes effectively cope with external pressures and maintain motivation levels. Vega-Díaz and González-García (2025) have shown that parenting styles significantly influence the development of mental toughness in young athletes, suggesting that external support systems can enhance athletes' ability to manage stress and prevent burnout. According to the self-determination theory (SDT), intrinsic motivation is significantly increased when an individual's sense of autonomy, competence, and relatedness are satisfied, and this increased motivation can alleviate burnout (Ryan and Deci, 2000). To better understand the psychological mechanisms underlying motivation and burnout, the SDT provides a comprehensive framework for understanding the dynamics of motivation. It encompasses sub-theories such as the basic psychological needs theory (BPNT) and the cognitive evaluation theory (CET). The BPNT emphasizes that the frustration of these needs can lead to maladaptive outcomes, such as emotional exhaustion and diminished self-worth, which are the hallmarks of athlete burnout. The CET expands on how external pressures, such as rewards or expectations, may either support or undermine intrinsic motivation, depending on their impact on autonomy and competence (Deci and Ryan, 1988). Adolescents, in particular, face unique challenges in balancing autonomy with social and parental expectations, which can exacerbate burnout if these expectations are not met. In this context, mental toughness can help athletes maintain motivation by supporting the fulfilment of these psychological needs in the face of external pressures (Cowden, 2017). In addition, life satisfaction, as an important mental factor, reflects an individual's subjective evaluation of his or her overall life situation and is closely related to mental health. Silvermark et al. (2008) stated that life satisfaction was a core indicator for assessing an individual's quality of life, and thus it is crucial to understand the effect of life satisfaction on athlete burnout in competitive sport (Silvemark et al., 2008). In the SDT framework, intrinsic motivation fulfilment and life satisfaction complement each other, and increased life satisfaction can effectively reduce the risk of athlete burnout. Therefore, this study aimed to investigate the mechanisms of life satisfaction and mental toughness in the role of sport motivation on athlete burnout. The findings would provide an empirical basis for coaches, sport psychologists and sport administrators to help them better understand the complexity of athlete burnout and provide more personalized and comprehensive psychological support to athletes.

Sport motivation, as a central component of the self-determination theory (SDT), plays a crucial role in the psychological state and behavioral performance of athletes (Grana et al., 2021). The SDT was developed by American psychologists, Deci and Ryan, in the 1980s and provides a comprehensive framework for understanding the antecedents and consequences of health-related behavioral motivation. The theory suggests that sport motivation can be categorized into intrinsic motivation, which is based on an individual's fun, sense of competence and appearance, and extrinsic motivation, which are closely linked to social and health factors. A study by Martins et al. (2022) confirmed the importance of sport motivation in predicting athlete burnout. Previous research has also shown that increasing athletes' participation in physical activity can improve their performance levels and psychological well-being (Biddle and Asare, 2011). Additionally, research has shown that by fostering key components of athletes' fun, competence, and appearance, their performance and overall life satisfaction can be improved (Mageau and Vallerand, 2003). The high correlation between sport motivation and psychological variables (e.g., mental toughness and psychological well-being) has been widely validated, which further strengthens the theoretical basis for examining the mediating effects of sport motivation on athlete burnout.

Life satisfaction, as an important indicator for individuals to evaluate their own quality of life, is closely related to their mental health and behavioral motivation. In the field of physical activity, life satisfaction not only reflects an individual's positive perception of the current state of life, but may also modulate the relationship between sport motivation and athlete burnout (Nicholls et al., 2015). According to the cognitive appraisal theory (Riley, 2016), individuals' appraisal of life events affects their emotional responses, and positive life satisfaction appraisals can help reduce feelings of stress and athlete burnout. Recent studies have further indicated that life satisfaction serves as a protective factor against psychological stress, enhancing athletes’ emotional resilience and sustaining their performance under high-pressure environments (Badura-Brzoza et al., 2022; Dwihandaka et al., 2024; Matijašević et al., 2025; Padmanabhanunni et al., 2023). The SDT further elucidates the relationship between life satisfaction and intrinsic motivation by suggesting that when individuals' basic psychological needs are satisfied, their intrinsic motivation is stronger, which helps them overcome challenges and reduces athlete burnout (Ryan and Deci, 2000). Therefore, fostering life satisfaction not only improves athletes’ mental toughness, but also reinforces their intrinsic motivation, offering a dual mechanism to alleviate athlete burnout.

Mental toughness, as it is distinct from psychological resilience, refers to an individual's ability to maintain performance and stay focused under pressure, rather than merely recovering from adversity (Denovan et al., 2022). Humińska-Lisowska et al. (2023) have further explored the genetic and personality factors associated with mental toughness, demonstrating that certain genetic polymorphisms, such as the rs4680 polymorphism of the COMT gene, are linked to personality traits that influence athletes' resilience and ability to cope with stress (Huminska-Lisowska et al., 2023). In sport, mental toughness is critical in helping athletes overcome mental barriers and stay motivated during training and competition. High mental toughness enables athletes to cope with challenges and minimize burnout. According to the mental toughness theory, athletes with high mental toughness can mobilize personal resources in the face of difficulties and use positive coping strategies to increase motivation and reduce burnout (Lou et al., 2014). Recent research further highlights the pivotal role of mental toughness in preventing performance decline and psychological exhaustion, especially in high-pressure environments (Hsieh et al., 2023; Liew et al., 2019). Additionally, as a stabilizing trait, mental toughness may moderate the relationship between sport motivation and athlete burnout, shaping the extent to which motivation impacts burnout.

**Figure 1:**
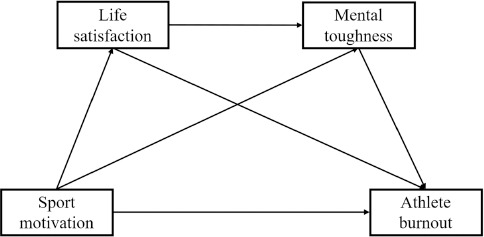
Proposed model.

Existing literature consistently demonstrates a significant correlation between sport motivation and burnout in athletes. However, the mechanisms underlying this relationship and its boundary conditions remain unexplored in a population of adolescent athletes. Therefore, this study aimed to investigate the mediating role of life satisfaction in the effect of sport motivation on burnout among adolescent athletes, as well as to examine the role of mental toughness as a moderating variable. Based on this, we proposed the following hypotheses: H1, sport motivation would directly predict athlete burnout; H2, life satisfaction would mediate the relationship between sport motivation and athlete burnout; H3, mental toughness would mediate the relationship between sport motivation and athlete burnout; and H4, life satisfaction and mental toughness jointly chain-mediated the relationship between sport motivation and athlete burnout. Figure 1 depicts the proposed model for the study.

## Methods

### Participants

This study used whole group sampling to administer questionnaires to adolescent athletes from several professional sports teams in the Shaanxi Province, the Henan Province and the Beijing City. The recruitment period was from the 11^th^ to the 28^th^ of September, 2023. Inclusion criteria were as follows: adolescent athletes aged 10 to 19 years old who volunteered to participate in this study (N = 682). On the other hand, athletes were excluded when they provided with incomplete questionnaire data (n = 44). A total of 682 questionnaires were distributed and 638 valid questionnaires were returned with a validity rate of 93.5%. The study was approved by the Academic Council of the Shaanxi Normal University (protocol code: 202216008; approval date: 13 April 2022). Informed consent was obtained from all adolescent athletes, their parents, and their coaches prior to the formal investigation. All participants and their guardians signed a written informed consent form.

The average age of the participants was 15.64 ± 2.28 years, of whom 345 (54.1%) were male athletes and 293 (45.9%) were female athletes. The sports practiced by participants and their distribution were as follows: athletics (20%), basketball (15%), table tennis (10%), wushu (8%), gymnastics (7%), wrestling (6%), kayaking (5%), weightlifting (5%), boxing (4%), taekwondo (3%) and archery (2%). On average, athletes reported training for 13.2 ± 3.5 hours per week. For more information on the participants, see Table 1.

**Table 1:** Demographic characteristics of participants (N = 638).

Gender		Number of cases
Male		345
Female		293
	**Variable**	**M ± SD**
**General Information**	Age (years)	15.64 ± 2.28
	Body height	168.11 ± 9.93
	Body mass	59.72 ± 18.48
	Training experience (years)	2.64 ± 1.58
	Training volume (hours per week)	13.2 ± 3.50
	**Category**	**Total sample N/%**
**Sports discipline**	athletics	20%
	basketball	15%
	table tennis	10%
	wushu	8%
	gymnastics	7%
	wrestling	6%
	kayaking	5%
	weightlifting	5%
	boxing	4%
	taekwondo	3%
	archery	2%

Note: M ± SD indicates mean ± standard deviation

### Measures

Sport motivation was measured using the measurement of motivation for physical activity-revised (MPAM-R) scale, which was developed by Deci et al. (2000) and simplified and developed by Chen et al. (2013) in Chinese based on the original scale. The present study directly adopted the simplified Chinese version of the MPAM-R developed by Chen et al. (2013). The scale consists of five dimensions of sport motivation: health, appearance, fun, ability, and socialization. The MPAM-R consists of 15 items, with three items for each dimension, and is rated on a 5-point Likert scale ranging from ‘none’ to ‘very strong’, with higher scores indicating higher levels of motivation. In this study, the simplified Chinese version of the scale showed sufficient reliability and validity, with a Cronbach's ɑ coefficient of 0.933, and reliability coefficients of the subscales ranging from 0.767 to 0.896.

The satisfaction with life scale (SWLS) developed by Espejo et al. (2021) was used in this study. The scale has been revised by Xiong and Xu (2009) and is a unidimensional tool consisting of five items on a 7-point Likert scale (1 = completely disagree, 7 = completely agree). Total scores for the SWLS ranged from 5 to 35, with higher scores indicating more life satisfaction. In this study, the Cronbach's alpha coefficient of the scale was 0.88, indicating good internal consistency. The Chinese version of the SWLS has also shown a high internal consistency (Cronbach's α = 0.91) and has been widely used in several studies (Liu et al., 2024; Tian and Wang, 2021).

Athlete mental toughness was measured with the sport mental toughness questionnaire (SMTQ). The questionnaire was originally developed by Sheard et al. (2009) and later revised by Wang et al. (2014). The questionnaire provides an overall sport mental toughness score as well as scores for three dimensions, i.e., self-confidence, constancy, and control. Total scores on the SMTQ range from 14 to 56, with higher scores indicating greater mental toughness. Previous research has shown that the SMTQ is a valid and reliable measurement tool (Sheard et al., 2009). In the current sample, the internal consistency of the questionnaire (14 items) was acceptable, with Cronbach’s α = 0.65.

Athlete burnout was measured using the athlete burnout questioners (ABQ) developed by Raedeke and Smith (2001), which consists of three dimensions, namely, emotional/physical exhaustion, a diminished sense of accomplishment, and a negative evaluation of the sport, with five entries for each dimension. The Chinese version of the ABQ, which has been translated and tested for its psychometric properties in previous research (Liu et al., 2022), was used in this study. The questionnaire was scored on a 5-point Likert scale ranging from 1 for “never” to 5 for “always”, with the 1^st^ and the 14^th^ entries being reverse scored. During the reliability test of this questionnaire, it was found that the factor loading of entry 1, “I am accomplishing many worthwhile things in sport”, did not meet the criterion of 0.4, thus it was decided to exclude it from the questionnaire. Ultimately, the total athlete burnout score was formed by summing the scores of the remaining 14 entries, and the higher the total score, the more severe the level of athlete burnout was reflected. The Cronbach's alpha coefficient for the total questionnaire in this study was 0.88 (14 items, α = 0.65).

### Design and Procedures

This study was cross-sectional. The questionnaires were distributed to the participants through an online platform and each participant received a unique link. To ensure the accuracy and completeness of the data, the questionnaires were completed under the supervision of coaches or researchers. The principles of confidentiality and anonymity were strictly adhered to in all data collection processes. The data collected were stored securely and only authorized personnel had access to it. Participants were assured that their responses would be used for research purposes only and that their identity would remain anonymous in any reports or publications of this study. All procedures followed were in accordance with the ethical standards of the responsible committee on human experimentation (institutional and national) and with the Helsinki Declaration of 1975, as revised in 2000. Informed consent was obtained from all participants involved in the study.

### Statistical Analysis

Initially, a questionnaire was used to assess all study variables, and the Harman's single factor test was applied to investigate the presence of the common method bias. Secondly, data did not conform to normal distribution, which was checked by the Kolmogorov-Smirnov test, and descriptive statistics were performed using the median of interquartile range [M(IQR)]. Subsequently, correlation analyses were performed using Spearman's rank correlation to explore the relationship among sport motivation, life satisfaction, mental toughness, and athlete burnout to test whether these variables were significantly related. Finally, the SPSS Process plugin was employed to perform mediation model testing and bootstrap analyses, in order to explore the mediating effects of life satisfaction and mental toughness in sport motivation and athlete burnout. In order to differentiate between the effects of gender, the study also performed separate analyses by gender.

## Results

### Common Method Deviation Test

The Harman single factor test was used to test for the common method bias. Results revealed that the first common component accounted for merely 27.5% of the total variance, below the critical criterion of 40% (Zhou and Long, 2004), suggesting a negligible common method bias in this research.

### Preliminary Analyses

As shown in Table 2, for both male and female adolescent athletes, sport motivation was significantly and negatively correlated with athlete burnout (males: r = −0.523; females: r = −0.491, *p* < 0.01), significantly and positively correlated with life satisfaction (males: r = 0.436; females: r = 0.331, *p* < 0.01), and mental toughness (males: r = 0.411; female: r = 0.543, *p* < 0.01). In addition, athlete burnout was significantly and negatively correlated with life satisfaction (male: r = −0.415; female: r = −0.436, *p* < 0.01) and mental toughness (male: r = −0.697; female: r = −0.600, *p* < 0.01), and life satisfaction was significantly and positively correlated with mental toughness (male: r = 0.430; female: r = 0.577, *p* < 0.01). These results provide an important basis for subsequent mediation effect analyses.

**Table 2:** Median, interquartile range and biased correlation analysis of each variable.

		Median (IQR)	1	2	3
**Male**	1. Sport motivation	0.23 (1.51)	1		
	2. Life satisfaction	0.23 (1.56)	0.436**	1	
	3. Mental toughness	−0.01 (1.29)	0.411**	0.430**	1
	4. Athlete burnout	−0.23 (1.10)	−0.523**	−0.415**	−0.697**
**Female**	1. Sport motivation	−0.02 (1.76)	1		
	2. Life satisfaction	−0.16 (1.56)	0.331**	1	
	3. Mental toughness	−0.01 (1.49)	0.543**	0.577**	1
	4. Athlete burnout	−0.03 (1.31)	−0.491**	−0.436**	−0.600**

Note: * indicates p < 0.05; ** indicates p < 0.01; IQR, interquartile range

### Sport Motivation Effects Athlete Burnout: A Chain Mediation Effect Test of Life Satisfaction and Mental Toughness

In order to explore the predictive relationships of sport motivation, life satisfaction and mental toughness on athlete burnout and to reveal potential gender differences, this study performed regression analyses for males and females separately. Sport motivation served as an independent variable, life satisfaction and mental toughness as mediating variables, and athlete burnout as a dependent variable. Control variables included age, height, body mass, the sport’s level, training volume and training experience. All variables were standardized and then tested for chain mediation effects using the bootstrap method (5000 samples) via the Process plug-in (Model 6) written by Igartua and Hayes (2021). The mediation effect was considered significant when the 95% confidence interval did not contain a zero, and conversely, the mediation effect was considered non-significant.

**Figure 2:**
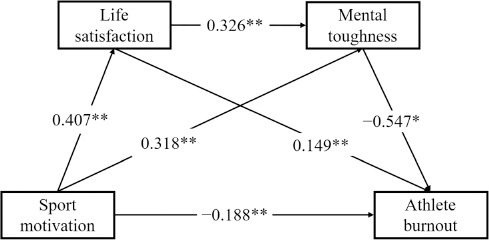
Chain mediation model of the relationship among sport motivation, life satisfaction, mental toughness, and athlete burnout in male adolescent athletes. Note: Solid lines are paths with significant effects; * p < 0.05; ** p < 0.01

**Figure 3:**
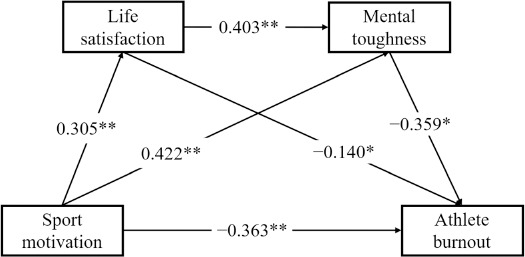
Chain mediation model of the relationship between sport motivation, life satisfaction, mental toughness, and athlete burnout in female adolescent athletes. Note: Solid lines are paths with significant effects* p < 0.05; ** p < 0.01

The chain mediated effects model is shown in Figures 2 and 3. The results of the regression analyses (Table 3) indicated, for male adolescent athletes, that sport motivation significantly and positively predicted life satisfaction (*β* = 0.407, *p* < 0.01). When sport motivation and life satisfaction jointly predicted mental toughness, both sport motivation (*β* = 0.318, *p* < 0.01) and life satisfaction (*β* = 0.326, *p* < 0.01) significantly positively predicted mental toughness. When sport motivation, life satisfaction, and mental toughness simultaneously predicted athlete burnout, sport motivation (*β* = −0.188, *p* < 0.01) and mental toughness (*β* = −0.547, *p* < 0.01) significantly negatively predicted athlete burnout, whereas life satisfaction was a significant positive predictor of athlete burnout (*β* = 0.149, *p* < 0.01). For female adolescent athletes, sport motivation significantly positively predicted life satisfaction (*β* = 0.305, *p* < 0.01). When sport motivation and life satisfaction jointly predicted mental toughness, both sport motivation (*β* = 0.422, *p* < 0.01) and life satisfaction (*β* = 0.403, *p* < 0.01) significantly positively predicted mental toughness. When sport motivation, life satisfaction, and mental toughness simultaneously predicted athlete burnout, sport motivation (*β* = −0.363, *p* < 0.01), life satisfaction (*β* = −0.140, *p* < 0.05), and mental toughness (*β* = −0.359, *p* < 0.01) all significantly negatively predicted athlete burnout.

**Table 3:** Regression analysis of sport motivation, life satisfaction, mental toughness and athlete burnout.

	Regression equation		R	R^2^	F	*β*	*t*	Confidence interval	
	Dependent Variable	Independent Variable						LLCI	ULCI
**Male**	Life satisfaction	Sport motivation	0.566	0.321	20.078**	0.407**	7.458	0.299	0.514
	Mental toughness	Sport motivation	0.541	0.293	15.050**	0.318**	5.175	0.197	0.438
		Life satisfaction				0.326**	5.100	0.199	0.451
	Athlete burnout	Sport motivation	0.742	0.551	38.743**	−0.188**	−3.843	−0.285	−0.092
		Life satisfaction				0.149**	0.051	0.048	0.249
		Mental toughness				−0.547**	−11.472	−0.641	−0.453
**Female**	Life satisfaction	Sport motivation	0.371	0.138	4.850**	0.305**	4.121	0.159	0.451
	Mental toughness	Sport motivation	0.730	0.532	29.426**	0.422**	7.492	0.312	0.533
		Life satisfaction				0.403**	7.471	0.296	0.509
	Athlete burnout	Sport motivation	0.709	0.503	22.793**	−0.363**	−5.321	−0.498	−0.229
		Life satisfaction				−0.140*	−2.136	−0.269	−0.011
		Mental toughness				−0.359**	−4.551	−0.514	−0.203

Table 4 presents the mediation effect paths of sport motivation on athlete burnout through life satisfaction and mental toughness and their significance test results among male and female adolescent athletes. For male adolescent athletes, the bootstrap 95% confidence interval for the total indirect effect did not contain 0, indicating a significant mediating effect. Specifically, there were two significant indirect paths: (1) the “sport motivation → mental toughness → athlete burnout” path, and its 95% confidence interval did not contain 0, indicating that mental toughness played a significant mediating role between sport motivation and athlete burnout (standardized effect value = −0.174, accounting for 46.52% of the total effect); (2) the “sport motivation → life satisfaction → mental toughness → athlete burnout” path, with the 95% confidence interval that did not contain 0, indicating that life satisfaction and mental toughness jointly had a significant chain mediating effect between sport motivation and athlete burnout (standardized effect value = −0.072, accounting for 19.25% of the total effect). Although the total indirect effect was significant, the confidence interval for the “sport motivation → life satisfaction → athlete burnout” pathway contained zero, indicating that life satisfaction did not mediate this pathway significantly. Since sport motivation was a significant direct prediction of athlete burnout, life satisfaction and mental toughness jointly played a partial mediating role between sport motivation and burnout. For female adolescent athletes, the bootstrap 95% confidence interval for the total indirect effect did not contain 0, indicating that significant mediating effects were present. There were three significant indirect pathways: 1) the “sport motivation → life satisfaction → athlete burnout” pathway with the 95% confidence interval that did not contain 0, indicating that life satisfaction had a significant mediating role in this pathway (standardized effect value = −0.043, accounting for 7.15% of the total effect); 2) the “sport motivation → mental toughness → athlete burnout” path, and its 95% confidence interval did not contain 0, indicating that mental toughness had a significant mediating role in this path (standardized effect value = −0.151, accounting for 25.12% of the total effect); 3) the “sport motivation → life satisfaction → mental toughness → athlete burnout” path with the confidence interval which did not contain 0, indicating that life satisfaction and mental toughness jointly had a significant chain mediating effect between sport motivation and athlete burnout (standardized effect value = −0.044, 7.32% of the total effect). Since sport motivation was a significant direct prediction of athlete burnout, life satisfaction and mental toughness played a partial mediating role between sport motivation and athlete burnout.

**Table 4:** Bootstrap analysis of the test of mediating effects of life satisfaction and mental toughness on the relationship between sport motivation and athlete burnout.

	Effects pathway	Indirect effect value	Bootstrap standard error	Boot LLCI	Boot ULCI	Relative effect %
**Male**	Total effect	−0.374	0.052	−0.476	−0.272	—
	Direct effect	−0.188	0.049	−0.285	−0.092	50.27%
	Total indirect effect	−0.186	0.035	−0.250	−0.110	49.73%
	Sport motivation → life satisfaction → athlete burnout	0.060	0.035	−0.002	0.135	16.04%
	Sport motivation → mental toughness → athlete burnout	−0.174	0.037	−0.245	−0.105	46.52%
	Sport motivation → life satisfaction → mental toughness → athlete burnout	−0.072	0.021	−0.116	−0.037	19.25%
	Total effect	−0.601	0.064	−0.726	−0.476	—
**Female**	Direct effect	−0.363	0.069	−0.499	−0.229	60.40%
	Total indirect effect	−0.238	0.048	−0.336	−0.146	39.60%
	Sport motivation → life satisfaction → athlete burnout	−0.043	0.022	−0.088	−0.002	7.15%
	Sport motivation → mental toughness → athlete burnout	−0.151	0.036	−0.225	−0.083	25.12%
	Sport motivation → life satisfaction → mental toughness → athlete burnout	−0.044	0.019	−0.089	−0.016	7.32%

## Discussion

This study explored the mechanisms of sport motivation on athlete burnout in adolescent athletes by introducing two variables: life satisfaction and mental toughness. A chain mediation model was constructed and validated to examine the complex relationship between sport motivation and athlete burnout. For male adolescent athletes, mental toughness played a significant independent mediating role between sport motivation and athlete burnout, while life satisfaction did not show an independent mediating effect. The hypothesized chain mediation model was confirmed for male athletes, validating hypotheses H1, H3, and H4, while H2 was not supported. For female adolescent athletes, both life satisfaction and mental toughness independently mediated the relationship between sport motivation and athlete burnout. The findings indicate that the chain mediation model was also applicable to female athletes, with hypotheses H1, H2, H3, and H4 being supported.

### Direct Role of Sports Motivation in Athlete Burnout

The results of the study showed a significant negative correlation between sport motivation and athlete burnout in both male and female athlete groups, i.e., the higher the level of sport motivation, the lower the level of athlete burnout. This suggests that not only is sport motivation a direct predictor of athlete burnout, but that this relationship shows consistency across both gender groups. This aligns with the core principles of the self-determination theory (SDT), which posits that motivation is a key predictor in predicting mental health and well-being (Ryan, 2000). Similarly, Zhang et al. (2010) also identified motivation as a predictor of athlete burnout, noting that athletes with low motivation had higher levels of burnout. In the context of sport, high motivation encourages athletes to engage more deeply in training, helping them better cope with challenges, thereby reducing burnout (Grana et al., 2021). Moreover, the direct predictive role of motivation highlights its potential value in preventing and intervening in athlete burnout. Increasing athletes' motivation levels can effectively predict and mitigate the risk of burnout. However, the results also suggest that other potential mediating factors should be considered when interpreting the relationship between sport motivation and burnout. Although previous studies, such as of Lonsdale et al. (2009), have demonstrated the relationship between intrinsic and extrinsic motivation and athlete burnout, the current study did not find significant mediating effects of these variables. This suggests that, under certain conditions or within specific populations, the effects of intrinsic and extrinsic motivation on burnout may not be as substantial as anticipated.

### Mediating Role of Mental Toughness in Athlete Burnout

The results of this study revealed the mediating role of mental toughness between sport motivation and athlete burnout in both male and female adolescent athletes. It was found that sport motivation not only directly predicted athletes' burnout levels, but also negatively and indirectly affected burnout by enhancing athletes' mental toughness. Specifically, adolescent athletes with higher mental toughness were able to demonstrate greater adaptability and resilience in the face of stress during training and competition, effectively reducing burnout. The results of this study fit with theories in the field of positive psychology, emphasizing the importance of mental toughness as a positive mental resource for individuals (Connaughton, 2010; Luthans and Youssef, 2007), and for the ability of adolescent athletes to remain positively adaptive in the face of challenges and adversity in training and competition. Middleton et al. (2004) revealed the multidimensional structure of mental toughness as a mental resource through in-depth, semi-structured interviews with 25 current and retired elite athletes, as well as eight veteran coaches from different sports. The data from these interviews summarized 12 key characteristics that together formed a framework for mental toughness, including: self-efficacy, mental self-concept, potential, task-specific attention, perseverance, task familiarity, personal bests, task value, goal commitment, positivity, stress minimization, and positive comparisons. These mental strengths enable adolescent athletes to have a more optimistic expectation that training and competition will be better and more effective in regulating their training state and mental state, which in turn alleviates athlete burnout. In the context of sport competition, athletes with higher mental toughness are more likely to adopt positive coping strategies, such as emotional regulation and problem solving, thereby mitigating the negative effects of burnout (Cowden, 2017).

### Chain Mediation of Life Satisfaction and Mental Toughness in the Role of Sport Motivation in Athlete Burnout

The findings suggest that male athletes' sport motivation has an inhibitory effect on athlete burnout through life satisfaction and mental toughness and forms a chain-mediated effect. Although life satisfaction correlates significantly with sport motivation and athlete burnout, it did not validate the role as an independent mediator variable in male athletes. Instead, life satisfaction indirectly affected athlete burnout through enhancing mental toughness, which plays a critical role in this process. Increasing life satisfaction and mental toughness can help male adolescent athletes better cope with training stress and reduce athlete burnout, especially in the context of multidimensional social support, where the interactive effect of the two is more significant. In female athletes, sport motivation similarly inhibited athlete burnout through life satisfaction and mental toughness, forming a chain mediating effect. However, unlike in male adolescent athletes, both life satisfaction and mental toughness exhibited independent mediating effects. Specifically, female adolescent athletes' life satisfaction directly influenced the reduction of athlete burnout, whereas mental toughness further mitigated athlete burnout by enhancing the ability to cope with adversity. This may be related to female adolescent athletes' higher sensitivity to subjective well-being and positive psychological resources, making them more inclined to rely on life satisfaction as a primary source of mental support in the face of training and competition stress (Tilga et al., 2021). This study highlights the importance of gender differences in the mechanisms influencing athlete burnout. For male adolescent athletes, it is suggested that individualized goal-setting and mental skills training to enhance mental toughness, along with strengthening team culture and social support to enhance their ability to cope with stress, could reduce the risk of burnout. For female adolescent athletes, intervention strategies could focus on enhancing life satisfaction, such as providing emotional support and enriching social interactions to help them maintain high subjective well-being during training and competition, while increasing mental toughness to better cope with challenges.

## Conclusions

This study emphasizes the pivotal role of sport motivation in reducing adolescent athlete burnout through both direct and indirect pathways. For female adolescent athletes, sport motivation directly reduces burnout and also affects burnout through three mechanisms: (1) a chain-mediated effect of life satisfaction and mental toughness, in which higher sport motivation enhances life satisfaction, which in turn enhances mental toughness, and ultimately reduces athlete burnout, (2) an independent mediating effect of life satisfaction, in which sport motivation directly enhances life satisfaction, which in turn reduces athlete burnout, and (3) an independent mediating effect of mental toughness, in which sport motivation directly enhances mental toughness and helps alleviate athlete burnout. For male adolescent athletes, only the chain-mediated effect of life satisfaction and mental toughness and the independent mediated effect of mental toughness were significant. These findings offer valuable theoretical insights into the multidimensional mechanisms of sport motivation on athlete burnout.
